# Trance and Possession Disorder With Dissociative-Psychotic Overlap: A Clinical Case Report

**DOI:** 10.7759/cureus.106654

**Published:** 2026-04-08

**Authors:** Christian David Galindo, Victor Agudelo, Ivan Ramirez Blanco, Karla Babativa Peñuela, Lesmer Galindo Ruiz

**Affiliations:** 1 Psychiatry, Corporación Universitaria Remington, Medellín, COL; 2 Psychiatry and Behavioral Sciences, E.S.E. Hospital Mental de Antioquia María Upegui, Medellín, COL; 3 Mental Health, Corporación Universitaria Remington, Medellín, COL; 4 Psychiatry and Behavioral Sciences, Corporación Universitaria Remington, Medellín, COL

**Keywords:** altered states of consciousness, cultural psychiatry, new-onset psychosis, public mental health, trance and possession disorder

## Abstract

Trance and possession disorder (TPD) is a complex and culturally influenced condition characterized by alterations in consciousness, identity, and behavior, often posing diagnostic challenges in clinical psychiatry. We report the case of a young woman who presented with a 12-hour history of visual and auditory hallucinations, disorganized speech, and aggressive behavior toward her partner, requiring emergency intervention and physical restraint.

Symptoms began with the perception of unusual noises and external presences in her home, reportedly shared by her partner, prompting religious consultation. Following this, the patient’s condition worsened, progressing to marked psychomotor agitation, aggression, and loss of behavioral control. The patient later described a trance-like state with a sense of loss of self and partial amnesia of the episode. There was no prior psychiatric history, substance use, or family history of mental illness.

The patient was managed with antipsychotic and benzodiazepine treatment, with subsequent clinical stabilization. This case highlights the diagnostic complexity of TPD, particularly its overlap with acute psychotic presentations and the influence of cultural context on symptom expression, emphasizing the need for a careful biopsychosocial approach in evaluation and management.

## Introduction

Trance and possession disorder (TPD) represents a rare yet clinically significant condition at the intersection of psychiatry, culture, and religion. It is characterized by episodes of altered consciousness in which individuals exhibit behaviors, speech, or actions that are incongruent with their usual personality, often posing substantial diagnostic and therapeutic challenges [1]. Dissociation, defined as a disruption in the integration of consciousness, memory, identity, perception, and motor control, constitutes the underlying psychopathological framework of these phenomena [2].

According to the Diagnostic and Statistical Manual of Mental Disorders, Fifth Edition, Text Revision (DSM-5-TR), dissociative identity disorder involves the presence of two or more distinct identity states accompanied by recurrent amnesia, whereas the International Classification of Diseases, Eleventh Revision (ICD-11) classifies TPD as a condition in which the individual’s identity is replaced by an external “possessing” entity, typically associated with a loss of voluntary control [3]. In contrast, the DSM-5-TR defines brief psychotic disorder as the presence of one or more psychotic symptoms, including delusions, hallucinations, disorganized speech, or grossly disorganized or catatonic behavior, with a sudden onset and a duration of at least one day but less than one month, followed by full return to premorbid functioning.

A defining feature of TPD is a marked narrowing or complete loss of awareness of the immediate environment, often manifesting as reduced responsiveness to external stimuli. These episodes may be accompanied by transient paralysis, altered consciousness, or stereotyped motor behaviors occurring outside voluntary control [4]. Clinically, presentations frequently adopt possession-form expressions, including voice alterations, glossolalia, abnormal movements, and behaviors interpreted as being driven by external forces [5]. While certain features of trance and possession states may overlap phenomenologically with psychotic symptoms, including externally attributed control and behavioral disorganization, these are better conceptualized within a dissociative framework when they occur in a culturally meaningful context, are transient in nature, and primarily involve alterations in identity and consciousness rather than a sustained disturbance of reality testing.

Despite its apparent rarity in clinical settings, possession states have been described across nearly all human societies. Cross-cultural evidence indicates that up to 90% of societies report institutionalized forms of altered states of consciousness, with many attributing these experiences to supernatural agents such as spirits, deities, or demonic entities [6]. The phenomenology and meaning of these experiences are deeply shaped by cultural and religious belief systems. In many contexts, religious practices - including prayer, exorcism, and ritual ceremonies - are employed as primary explanatory and therapeutic frameworks [7]. While culturally meaningful, these interpretations may, in certain cases, reinforce symptom expression, delay psychiatric evaluation, and contribute to diagnostic complexity.

Importantly, from the perspective of the DSM-5-TR psychotic spectrum, certain manifestations observed in TPD, such as externally attributed control, behavioral disorganization, or perceptual disturbances, may overlap phenomenologically with psychotic symptoms. However, these features are typically transient, contextually coherent, and strongly embedded within culturally meaningful frameworks, which distinguishes them from primary psychotic disorders. This distinction reflects a broader conceptual tension between biological psychiatry, which emphasizes neurobiological mechanisms underlying psychosis, and cultural psychiatry, which highlights the role of sociocultural context in shaping symptom expression and interpretation.

Epidemiological data suggest that dissociative disorders, particularly dissociative identity disorder, have a prevalence of approximately 1.1% to 1.5% in the general population, rising to nearly 5% in clinical settings [8]. These conditions are strongly associated with early-life trauma, including physical and sexual abuse and emotional neglect, which may lead to alterations in functional brain connectivity and the development of dissociative coping mechanisms. Importantly, dissociative disorders are associated with high morbidity, including elevated rates of self-injurious behavior and suicide attempts [9].

In this context, we present the case of a patient whose acute clinical presentation, initially interpreted within a religious framework as possession, revealed a complex neuropsychiatric condition characterized by dissociative-psychotic overlap, highlighting the diagnostic challenges at the interface of cultural meaning and psychiatric classification.

## Case presentation

A 26-year-old woman with no significant past medical or psychiatric history was brought to the emergency department with a 12-hour history of acute behavioral disturbance, characterized by visual and auditory hallucinations, disorganized speech, and aggressive behavior toward her partner. The patient had no history of substance use, no known family history of psychiatric disorders, and no reported history of significant psychological trauma. Prior to the onset of symptoms, she maintained adequate social and occupational functioning, with no evidence of functional impairment. Due to the severity and rapid escalation of symptoms, emergency services were required. The patient exhibited marked hostility and physical aggression, necessitating both pharmacological sedation and physical restraint, with the involvement of a greater number of personnel than typically required to ensure safe containment.

According to the patient and her partner, symptoms began four days prior with the perception of unusual noises and the sensation of external presences within their home, which progressively intensified, with a marked worsening during the last 24 hours. Both reported these experiences, prompting them to seek religious intervention. Notably, the patient exhibited a rapid clinical deterioration immediately following the arrival of a priest and the initiation of prayer, developing intense headache, vomiting, behavioral disorganization, and a subjective sense of loss of control over her body.

During this episode, the patient entered a trance-like state characterized by incoherent speech, destructive behavior, and marked behavioral disinhibition. She displayed targeted aggression toward the priest, unexpectedly verbalizing his name without prior knowledge. She destroyed religious objects, including devotional figures and rosaries, and physically assaulted the priest using a Bible, causing a head injury. This escalation led to the involvement of police and emergency services. The patient later reported partial amnesia of the event.

In the emergency department, the patient underwent initial stabilization with pharmacological management and close clinical monitoring. On mental status examination at the time of admission, she was markedly agitated, with disorganized speech, perceptual disturbances including visual and auditory hallucinations, and impaired behavioral control. Thought processes were disorganized, and both insight and judgment were significantly compromised; reality testing was impaired during the acute episode. No formal psychometric assessments were conducted due to the severity of the presentation.

A comprehensive physical examination, including assessment of vital signs, was performed and revealed no significant abnormalities. A complete diagnostic workup was undertaken to exclude organic etiologies. Laboratory investigations, including metabolic panel, thyroid function tests, infectious screening, and toxicology testing, were within normal limits. Relevant laboratory findings are summarized in Table 1.

**Table 1:** Relevant laboratory findings VDRL: Venereal Disease Research Laboratory; HIV: human immunodeficiency virus; THC: tetrahydrocannabinol

Category	Test	Patient’s Result	Reference Range/Interpretation
Toxicology screening	Amphetamines	Negative	Negative
	Cocaine	Negative	Negative
	Cannabis (THC)	Negative	Negative
	Opiates	Negative	Negative
	Benzodiazepines	Negative	Negative
	Barbiturates	Negative	Negative
	Methadone	Negative	Negative
	Phencyclidine (PCP)	Negative	Negative
	Tricyclic antidepressants	Negative	Negative
	Synthetic cannabinoids	Negative	Negative
Infectious screening	VDRL	Non-reactive	Non-reactive
	HIV	Negative	Negative
	Hepatitis B surface antigen (HBsAg)	Negative	Negative
Renal function	Creatinine (mg/dL)	0.7	0.6-1.2
	Blood urea nitrogen (BUN, mg/dL)	12.9	7-20
	Urea (mg/dL)	27.7	15-40
Electrolytes	Sodium (Na, mEq/L)	143.4	135-145
	Potassium (K, mEq/L)	4.13	3.5-5.0
	Magnesium (Mg, mg/dL)	2.05	1.7-2.4
	Chloride (Cl, mEq/L)	105.6	98-107
Liver function	Total bilirubin (mg/dL)	0.21	0.2-1.2
	Direct bilirubin (mg/dL)	0.09	0.0-0.3
	Indirect bilirubin (mg/dL)	0.12	0.2-0.8
Thyroid function	Thyroid-stimulating hormone (TSH, mIU/L)	1.22	0.5-5.0
Hematology	Hemoglobin (g/dL)	15	12-16 (female)
	Hematocrit (%)	46.4	36-46
	White blood cell count (×10^9^/L)	10.85	4.5-11.0
	Neutrophils (%)	64	40-70
	Platelets (×10^9^/L)	499	150-450

**Figure 1:**
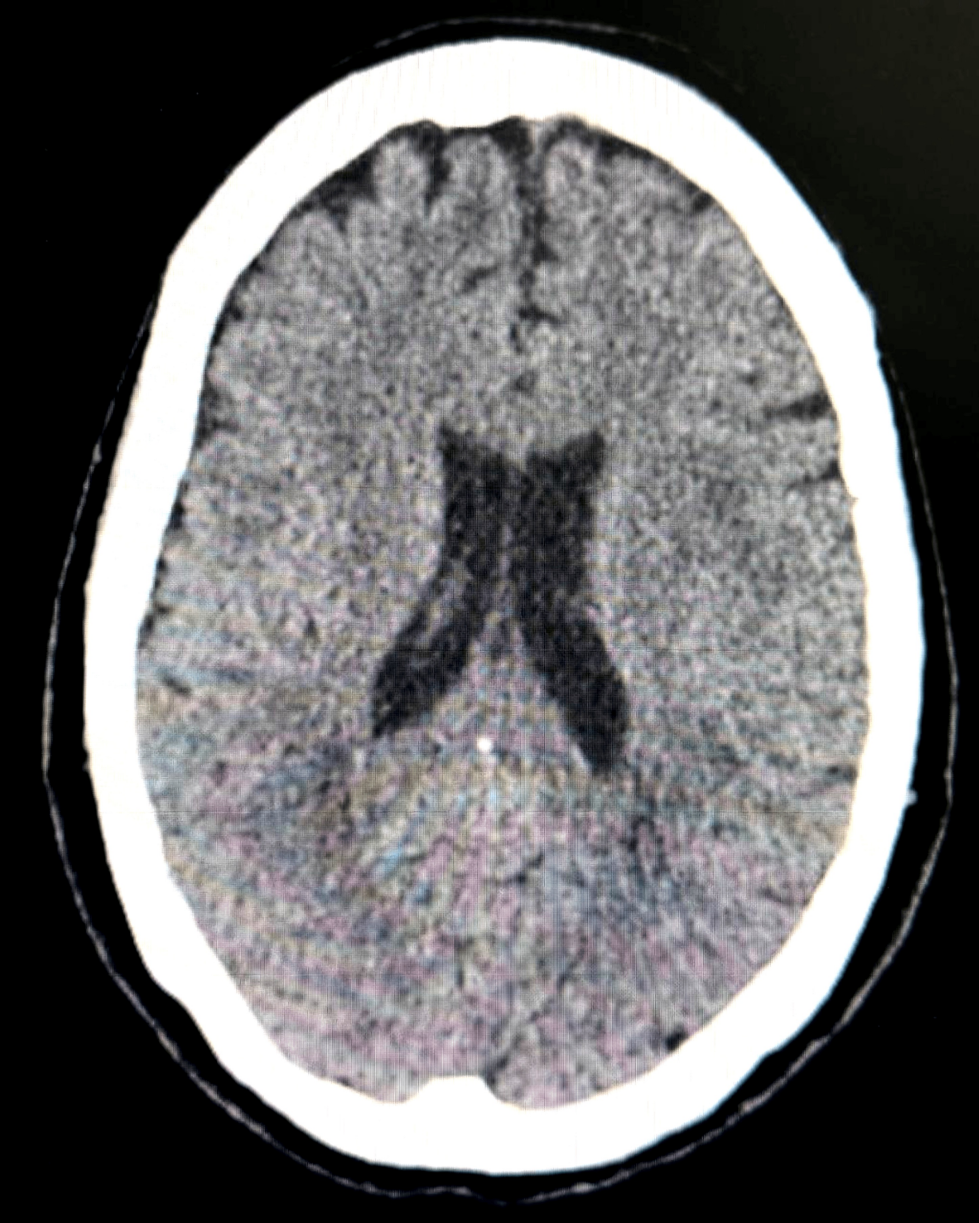
Non-contrast CT of the brain Axial non-contrast CT showing preserved gray-white matter differentiation and symmetric ventricular system, without evidence of acute hemorrhage, mass effect, or ischemic lesions.

Initial neuroimaging with non-contrast computed tomography (CT) of the brain revealed no evidence of acute ischemia, hemorrhage, or space-occupying lesions, failing to account for the severity of the patient’s neuropsychiatric presentation (Figure 1).

Neurological assessment, including formal evaluation by the neurology team, demonstrated no focal deficits or evidence of underlying structural or functional neurological pathology.

Initial management included pharmacological sedation administered in the emergency setting to control severe agitation and aggressive behavior. The patient was treated with olanzapine 10 mg every 12 hours, with good clinical tolerance, followed by close clinical observation.

Given the atypical presentation and fluctuating clinical course, the patient was admitted for observation. Notably, she remained off antipsychotic treatment for four days, during which a progressive and spontaneous clinical improvement was observed, with complete resolution of psychomotor agitation and perceptual disturbances, and no recurrence of active psychotic symptoms.

During hospitalization, a hospital-affiliated priest conducted a structured religious intervention consisting of guided prayers. Following this intervention, the patient reported a marked subjective improvement in her condition. However, she continued to express significant fear of returning to her home environment, which she associated with the onset of symptoms. Consequently, she and her partner decided to relocate to a different residence. At discharge, the patient was clinically stable, euthymic, with preserved insight and no evidence of ongoing psychotic or dissociative symptoms. She was referred for outpatient psychiatric follow-up and longitudinal monitoring.

This case illustrates a complex acute neuropsychiatric presentation characterized by prominent dissociative features within a culturally and religiously mediated context. It underscores the diagnostic challenges in distinguishing between acute psychotic disorders, dissociative phenomena, and trance and possession states, highlighting the need for a comprehensive, culturally informed, and multidisciplinary approach to assessment and management.

## Discussion

Trance and possession states represent a central challenge for contemporary psychiatry, as they lie at the intersection of psychopathology, culture, and meaning-making processes. These phenomena challenge strictly biomedical models, which tend to localize mental illness solely within the brain, often overlooking the symbolic and sociocultural determinants of subjective experience [10]. However, contemporary psychiatric frameworks increasingly emphasize the need to integrate biological, psychological, and cultural perspectives in understanding complex neuropsychiatric presentations.

Within this integrative framework, TPD can be conceptualized within the dissociative spectrum, where symptoms reflect culturally mediated expressions of distress. Cross-cultural evidence suggests that these states function as an “idiom of distress,” whose phenomenology is shaped by sociocultural context. Emerging models describe a progression from “passive influence” experiences, such as perceived external control or the sensation of presence, toward more structured possession states involving altered consciousness and behavioral disorganization, consistent with the clinical course observed in this case [11].

The concept of agency provides a unifying framework for understanding these phenomena. Possession may be interpreted as a transient disruption in the sense of authorship over one’s thoughts and actions, whereby individuals experience their behavior as being controlled by an external force. Importantly, this alteration cannot be understood in isolation from cultural context, which provides the symbolic narratives that shape and give meaning to the experience [12]. From a medical anthropological perspective, possession states have been conceptualized as mechanisms for expressing distress and regulating individual and social tensions, allowing for the integration of cultural and psychiatric models within a biopsychosocial framework [13].

Nevertheless, the clinical presentation also raises important diagnostic considerations within the psychotic spectrum. Features such as visual and auditory hallucinations, behavioral disorganization, and aggression are traditionally associated with primary psychotic disorders, including brief psychotic disorder. This condition, characterized by an acute onset and full remission within a short duration, represents a relevant differential diagnosis in this case. Importantly, the spontaneous remission observed here should be interpreted with caution, as it is not specific to dissociative disorders and may also occur in acute psychotic conditions.

Additionally, the report of shared perceptual experiences between the patient and her partner introduces the possibility of shared psychotic phenomena (folie à deux). However, the absence of a sustained shared delusional system, the asymmetry in symptom severity, and the rapid resolution of symptoms argue against this diagnosis as the primary explanatory framework.

Despite this overlap, several features supported a dissociative conceptualization. These include the marked alteration in identity and sense of control, the strong cultural and religious embedding of the experience, the presence of partial amnesia, and the transient, non-persistent course without residual psychotic symptoms or functional decline. These characteristics align more closely with dissociative and trance-related phenomena as described in ICD-11. Furthermore, similar cases reported in the literature describe acute dissociative states with possession-form presentations in culturally mediated contexts, often posing diagnostic challenges due to their overlap with psychotic symptomatology.

The temporal association between religious intervention and symptom exacerbation in this case suggests a role for suggestion, emotional arousal, and culturally shaped expectations in modulating symptom expression. At the same time, the subsequent clinical improvement, while notable, should not be interpreted as pathognomonic of a dissociative process, but rather as part of a broader clinical trajectory requiring cautious interpretation [14].

This case also highlights the role of stigma and belief systems in shaping illness trajectories. The initial reliance on religious explanations and the shared perceptual experiences reported by the partner illustrate how cultural frameworks can reinforce symptom expression and delay access to psychiatric care.

Future research should focus on better delineating the boundaries between dissociative and psychotic phenomena, particularly in culturally diverse settings. There is a need for integrative models that incorporate neurobiological, psychological, and sociocultural dimensions, as well as longitudinal studies exploring the evolution and outcomes of such presentations. Improved diagnostic frameworks may help reduce misclassification and enhance culturally sensitive clinical care.

Overall, this case underscores the need for an integrative clinical approach that moves beyond reductionist models, incorporating cultural, symbolic, and relational dimensions into psychiatric assessment and management. Bridging clinical and cultural perspectives not only enhances diagnostic accuracy but also facilitates more contextualized, effective, and patient-centered interventions [15].

## Conclusions

TPD represents a diagnostically complex condition situated at the interface of dissociative psychopathology, cultural meaning systems, and acute neuropsychiatric presentations. This case illustrates the critical importance of a rigorous and integrative diagnostic approach, particularly in contexts where dissociative and psychotic features coexist and may be phenomenologically indistinguishable. Although the patient exhibited prominent psychotic-like symptoms, including hallucinations and behavioral disorganization, the overall clinical profile - marked by alterations in identity and sense of agency, culturally congruent symptom expression, partial amnesia, and a transient course with full recovery and no residual impairment - supports a dissociative conceptualization rather than a primary psychotic disorder.

This case underscores the necessity of carefully differentiating dissociative states from psychotic disorders, as diagnostic misclassification may lead to suboptimal management and misinterpretation of the underlying condition. Importantly, it highlights the role of cultural context not as a confounding factor, but as a central element in symptom formation and clinical interpretation. Ultimately, adopting a biopsychosocial and culturally informed framework allows for more precise diagnostic formulation, reduces the risk of diagnostic overshadowing, and supports the delivery of more effective, patient-centered care in complex acute presentations.
